# Controlled Release of Flavor Substances from Sesame-Oil-Based Oleogels Prepared Using Biological Waxes or Monoglycerides

**DOI:** 10.3390/foods10081828

**Published:** 2021-08-07

**Authors:** Min Pang, Lulu Cao, Shengmei Kang, Shaotong Jiang, Lili Cao

**Affiliations:** 1 School of Food Science and Bioengineering, Hefei University of Technology, Hefei 230009, China; pangmin@hfut.edu.cn (M.P.); 2018111236@mail.hfut.edu.cn (L.C.); 2020171424@mail.hfut.edu.cn (S.K.); jiangshaotong@163.com (S.J.); 2 Key Laboratory for Agricultural Products Processing of Anhui Province, Hefei 230009, China

**Keywords:** sesame oil, gelation, oleogels, controlled volatile release

## Abstract

The flavor substances in sesame oil (SO) are volatile and unstable, which causes a decrease in the flavor characteristics and quality of SO during storage. In this study, the effect of gelation on the release of flavor substances in SO was investigated by preparing biological waxes and monoglycerides oleogels. The results showed that the release of flavor substances in SO in an open environment is in accordance with the Weibull equation kinetics. The oleogels were found to retard the release of volatiles with high saturated vapor pressures and low hydrophobic constants in SO. The release rate constant k value of 2-methylpyazine in BW oleogel is 0.0022, showing the best retention effect. In contrast, the addition of gelling agents had no significant retention effect on the release of volatiles with low saturated vapor pressures or high hydrophobic constants in SO, and even promoted the release of these compounds to some extent. This may be due to the hydrophilic structural domains formed by the self-assembly of gelling agents, which reduces the hydrophobicity of SO. This work provides a novel approach for retaining volatile compounds in flavored vegetable oils. As a new type of flavor delivery system, oleogels can realize the controlled release of volatile compounds.

## 1. Introduction

Sesame (*Sesamum indicum* L.) oil (SO), a traditional vegetable oil used for seasoning in China, has a strong and unique flavor and is rich in nutrients. The flavor substances in SO are formed by a series of Maillard reactions, oxidation reactions, and caramelization reactions during processing, which produce furans, pyrazines, aldehydes, alcohols, ketones, and pyrroles. In addition, SO can have an unsaturated fatty acid content of more than 80% [1] and is rich in phytosterols, tocopherols, lignans, vitamins, and trace elements such as iron, zinc, and copper [2,3]. Owing to its flavor and nutritional value, SO has become widely used for seasoning in China and is popular with consumers. However, the flavor substances in SO undergo osmotic diffusion during processing and storage owing to their volatility and poor stability, which leads to flavor loss and quality degradation. The diffusion release process of small flavor molecules follows the zero-order release kinetic equation, first-order release kinetic equation, Higuchi equation, or Weibull equation [4,5,6,7]. Under the action of a concentration gradient, small flavor molecules diffuse through the vegetable oil matrix and volatilize on the outer surface. Because SO has a strong flavor, the loss of flavor substances is of particular significance. Therefore, research on effective SO flavor preservation technologies is important to enhance the nutritional function of SO and promote the modernization of traditional flavored oils.

In recent years, gel formation for vegetable oil solidification and corresponding application technologies has become a research hotspot [8]. Gels of vegetable oils are formed by melt-cooling and dispersing a gelling agent in vegetable oils. Subsequently, the system is gelled by forming a thermally reversible three-dimensional network structure through non-covalent physical bonds, which prevent the flow of vegetable oils [9]. Oleogels are as strong and malleable as solid fats and their use can radically reduce the content of trans and saturated fats in food. Owing to these advantages, oleogels are considered a healthy fat type that can replace traditional solid fats in various applications [10,11]. The n-alkanes are important for the gelation of vegetable oils. Studies conducted on vegetable oils found that the n-alkane composition varied depending on the botanical origin [12]. Sesame oil [13], sunflower oil [14], olive oil [15,16], palm oil [17], peanut oil [14] and linum seed oil [18] all have specific patterns of n-alkane carbon chain length composition and content. For instance, the carbon atom chain of n-alkanes in olive oil [16] ranges from 24 to 260 mg/kg, and the carbon atom chain of n-alkanes in sesame oil [13] ranges from 22 to 82 mg/kg. Currently, oleogels are typically prepared using small-molecule gelling agents such as γ-oryzanol, sterols, monoglycerides, fatty acids, and natural waxes. Monoglycerides (GMS) refers to a series of surfactants produced by interesterification of fats or oils with glycerol. GMS is one of the commonly used gelling agents in food production, which can form a crystalline network in oils and then form elastic gels [19]. Further studies on the controlled release of volatile substances by grease gels have revealed that wax-based oleogels can effectively control and slow the release of flavor substances, thus providing a promising and adjustable delivery strategy for flavor compounds [20,21]. However, research on oleogels has mainly focused on the gelation mechanism, physicochemical properties, nutritional function, and applications. In contrast, there is insufficient knowledge about the effect of gel structures and intermolecular interaction mechanisms on the release of small flavor molecules. In particular, the effect of gelation on the mechanism of controlled flavor substance release from SO has not been investigated.

In this study, sesame-oil-based oleogels were prepared using biological waxes and monoglycerides as gelling agents, and the controlled release of volatile flavor substances was investigated after gelation. The physical properties, microstructures, thermodynamic properties, and crystal morphologies of the oleogels were characterized using texture analysis, polarized light microscopy, differential scanning calorimetry (DSC), and X-ray diffraction (XRD). The release kinetics of the characteristic flavor substances in SO was studied in the oleogels to clarify the effect of SO self-assembly behavior on the controlled release of small flavor molecules. These results can aid in establishing controllable flavor delivery systems and technologies for maintaining the nutritional value and flavor of SO.

## 2. Materials and Methods

### 2.1. Materials and Chemicals

Candelilla (*Euphorbia antisyphillitica*) wax (CLW), rice bran (*Oryza sativa* L.) wax (RBW), carnauba (*Copernicia prunifera* (Mill.) H.E. Moore) wax (CRW) and beeswax (*Apis mellifera*) (BW) were purchased from Changge City, Yi Heng Jian apiculture, Ltd. (Henan, China). Glycerol monostearate (GMS) was purchased from Shanghaiyuanye Bio-Technology Co., Ltd. (Shanghai, China). Standards of the flavor compounds were obtained from Sigma Chemical Co., Ltd. (Shanghai, China). All organic reagents used in the experiments were of analytical grade and obtained from Sinopharm Holdings Ltd. (Shanghai, China). Sesame oil was obtained from a local market (Hefei, China).

### 2.2. Preparation of Oleogels

The gelling agents (CLW, RBW, CRW, BW or GMS) and SO were accurately weighed and placed in a clean, dry glass container, which was then covered with cling film. The samples were heated with stirring (magnetic stirrer, 250 rpm) for 15 min (90 °C) to completely dissolve the gelling agents, after which time, the mixtures were deposited onto a receptacle suitable for analysis. Then, the mixtures were cooled to room temperature (~25 °C). All characterizations were performed 24 h after gel preparation. Testing was carried out on triplicate sample sets and all gel concentrations were reported as percentage (wt.%). wt.% (weight percent) = [g solute/(g solute + g solvent)] × 100, so 5 wt.% CLW in SO should be: 5 g CLW in 95 g SO.

### 2.3. Determination of Critical Concentrations

Sesame oil and gelling agent were heated in a water bath until the gelling agent was completely dissolved (90 °C, 15 min), and poured into 10 mL glass vials (20 mm diameter × 50 mm length). The test was started with 1% (wt.) of the additive and subsequently increased the dosage to 10% in 1% increments. This series of samples was stored at room temperature (~25 °C) for 24 h, and the sample was inverted to observe whether there was gravity flow. In this way, the minimum gelling agent concentration without flowing was the critical concentration. The experiment was repeated three times for each test.

### 2.4. Microscopy

The crystal morphology of the oleogels were observed using a MP 30 polarized light microscope (Mshot, Guangzhou, China). 20 µL of the molten sample was gently spread on a preheated slide and covered with a coverslip. The samples were stored at room temperature (~25 °C) for 24 h and the microstructure of all the samples was observed and the crystalline morphology of the samples was photographed.

### 2.5. Texture Analysis

The firmness and stickiness of gel samples were measured using a texture analyzer TA-XT plus (Stable Microsystems, Surrey, UK) equipped with a 12.7 mm cylindrical probe (P/0.5). All samples were measured after 24 h of preparation at room temperature (~25 °C). Texture analyses were performed by inserting the probe into the sample at a speed of 1 mm/s to a maximum penetration depth of 10 mm and pull the probe out at a speed of 10 mm/s. The firmness and stickiness were calculated using Texture Exponent v.6.1.16.0 software (Stable Microsystems).

### 2.6. Thermal Properties

To investigate the melting and crystallization behaviors of the samples, DSC thermograms were obtained using a Q2000 differential scanning calorimeter (TA Instruments, New Castle, DE, USA). Samples with a mass of ca. 10–15 mg were sealed in aluminum discs and heated from room temperature (~25 °C) to 100 °C at a rate of 20 °C/min, held at 100 °C for 5 min to remove crystallization memory, then cooled to 0 °C at a rate of 10 °C/min and held for 5 min to fully crystallize, and finally heated to 100 °C at a rate of 10 °C/min. Meanwhile, empty aluminum discs were used as controls in the experiment. The onset crystallization temperature (T_g_, °C), peak crystallization temperature (T_c_, °C) and crystalline enthalpy (ΔH_c_, J/g) during the cooling phase and peak melting temperature (T_m_, °C) and melting enthalpy (ΔH_m_, J/g) during the heating phase were calculated using TA Universal Analysis software (TA Instrument, USA).

### 2.7. XRD Measurements

An X-ray diffractometer (PANalytical B.V., Almelo, The Netherlands) was used to determine the crystal structure of samples using a copper X-ray tube (λ = 1.54 Å) with an operating voltage of 40 kV and a current of 40 mA as the X-ray source. Samples were prepared on a slide, and the sample surface was smoothly treated by rotating the slide around an axis perpendicular to the plate surface. Diffraction patterns were measured by scintillation detector with a scanning speed of 4°/min and a scanning range of 5.0°–50° (2θ) at room temperature (~25 °C). The X-ray diffraction patterns of the samples were analyzed using MDI Jade 6.0 software (Materials Data Ltd., Livermore, CA, USA).

### 2.8. Determination of Cumulative Release Rate of Flavor Compounds

Sesame oil (5 g) or an oleogel sample with 8 wt.% gelling agent (5.435 g) was accurately weighed and placed in a 20 mL headspace vial (to equalize the SO content). The vial then was left open and stored at 25 °C for 60 d. The flavor compounds in the sample were analyzed by GC-MS after 0, 1, 3, 4, 5, 7, 9, 11, 13, 16, 19, 21, 23, 27, 32, 37, 42, 47, 54, and 60 d. Before analysis, the lid was sealed (silicone/polytetrafluoroethylene seal, La-Pha-Pack GmbH, Germany) and the sample was equilibrated in a constant temperature water bath at 60 °C with magnetic stirring for 20 min. An activated SPME fiber (Supelco, Bellefonte, PA, USA) was inserted into the headspace vial. After adsorption for 50 min, the SPME fiber was inserted into the GC inlet, where thermal desorption at 250 °C was allowed to proceed for 6 min before data collection. Three parallel samples were prepared for each time point, and each sample was tested only once.

GC-MS analysis was performed using an Agilent 6890 GC-5975I MS instrument (Agilent, Santa Clara, CA, USA) equipped with a DB-5MS capillary column (60 m length × 1 mm inner diameter, 0.32 µm film thickness). Analyses were carried out in splitless mode with helium as the carrier gas at a flow rate of 1.7 mL/min. The oven temperature was initially held at 50 °C for 2 min, increased to 220 °C at a rate of 4 °C/min, and then held at 220 °C for 10 min. The mass spectrometry interface and ion source temperatures were both 250 °C. Ionization was performed in electron impact mode at 70 eV. Chromatograms were collected in full scan mode with a mass scan range of m/z 30–550.

The cumulative release rate, Q, was used to describe the dynamic release process of the flavor compounds. The Q values of 28 flavor compounds in SO were calculated using the following equation: Q = (C_0_ − C_t_)/C_0_ × 100%, where C_0_ is the initial concentration of the flavor compound and C_t_ is the concentration of the flavor compound on day t (t, the storage time). Volatile compounds were qualitatively analyzed by standard compounds, mass spectra in the database (NIST18. LIB) and retention index. The external standard method was used for the quantitative analysis of flavor compounds. Using a series of working standard solutions, the peak area corresponding to each standard in the mixture was determined at varying concentrations, and standard curves were constructed. The concentrations of the flavor compounds (μg/kg) in the SO or oleogel samples were then determined from the standard curve using the corresponding peak areas, which were the average values from three measurements.

### 2.9. Data Analysis

The analysis of variance (ANOVA) and significant differences were calculated by using SPSS 18.0 statistical software (SPSS Inc, Chicago, IL, USA). Sample preparation and analyses were performed in triplicate, and the results were expressed as mean ± standard deviation (x ± SD). Data analysis and plotting were performed by using Origin 9.0 (OriginLab, Northampton, MA, UK) and MATLAB R2018a (MathWorks, Neddick, MA, USA.

## 3. Results and Discussion

### 3.1. Determination of Critical Concentration

**Figure 1:**
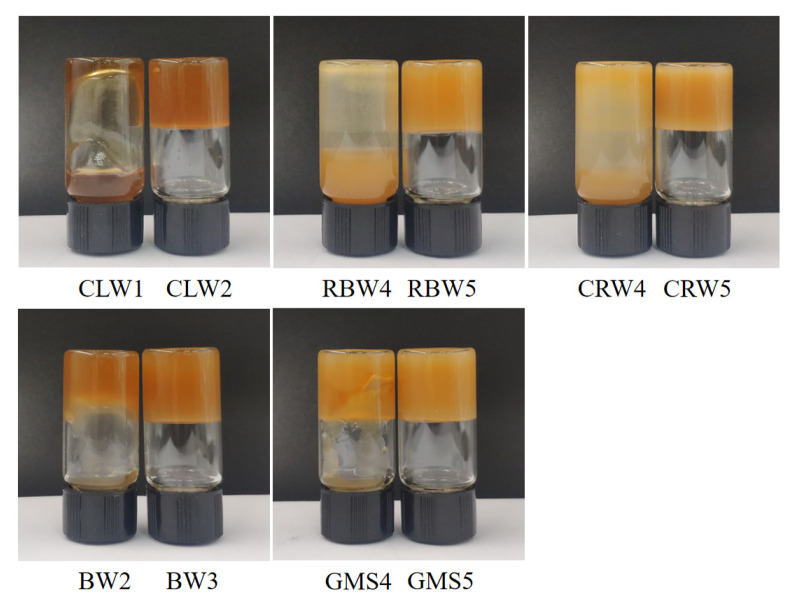
Determination of critical concentration of different types of oleogel (wt.%). CLW, candelilla wax; RBW, rice bran wax; CRW, carnauba wax; BW, beeswax; GMS, glycerol monostearate.

As shown in Figure 1, CLW had the best gelation effect among the investigated samples, forming a gel with SO at an addition level of 2 wt.%. Table S1 indicates the minimum quantity (wt.%) required for SO gelation and composition of gelling agents. As previously reported [22,23,24], CLW predominantly consists of 49–50% n-alkanes (C29–C33), 20–29% esters of acids and alcohols with even-numbered carbon chains (C28–34), 12–14% resins (mainly triterpenoid esters), and 7–9% free acids. Abdallah and Weiss [25] showed that n-alkanes are the structurally simplest organic gelling agents and that the stability of a gelled oil increases as the n-alkane chain length increases. Morales-Rueda, Dibildox-Alvarado, Charó-Alonso, Weiss and Toro-Vazquez [23] investigated the thermal and oil-holding properties of the main component of CLW, n-tridodecane (C32), when added to safflower oil to prepare oleogels. They found that C32 oleogels have a better self-assembly ability than CLW oleogels, which indicates that the n-alkanes in CLW have a better gelation ability than the minor components. Thus, the high content of C29–C33 n-alkanes in CLW (49–50%) may be responsible for the better gelling ability of CLW than that of other waxes. BW is a mixture of odd-numbered hydrocarbons (mainly heptacosane), even-numbered hydrocarbons, odd-numbered monounsaturated hydrocarbons, fatty acid esters combined with long-chain alcohols, and free wax acids [26]. BW was also shown to be a good gelling agent for SO, with the formation of a stable gel observed at a concentration of 3 wt.%. Esters derived from long-chain saturated fatty acids (C24 and C22) and long-chain saturated fatty alcohols (C28–C34) are the major components in RBW [27]. In contrast to the results reported by Hwang, Kim, Singh, Winkler-Moser and Liu [24], which showed good gelation of soybean oil with RBW, the critical concentration of RBW for SO gelation was 5 wt.%. This difference in behavior may be due to variations in the wax composition with season, region, and purity, indicating that knowledge of the exact composition is important for understanding the gelation properties of a wax. The main components of CRW are 84–85% aliphatic and aromatic esters (including 40% aliphatic esters, 13% ω-hydroxy esters, and 8% cinnamic aliphatic diesters) [28]. The critical concentration of CRW was 5 wt.% owing to the weak gelation ability of the mixture of aliphatic esters and p-hydroxycinnamic aliphatic diesters. GMS, consisting of 84.5% monostearin and 12.2% monopalmitin, is one of the most commonly used gelling agents. The critical concentration of GMS in SO was 5 wt.%. Thus, the gelation ability of GMS was similar to that of RBW and CRW but weaker than that of CLW and BW.

### 3.2. Microscopic Analysis

**Figure 2:**
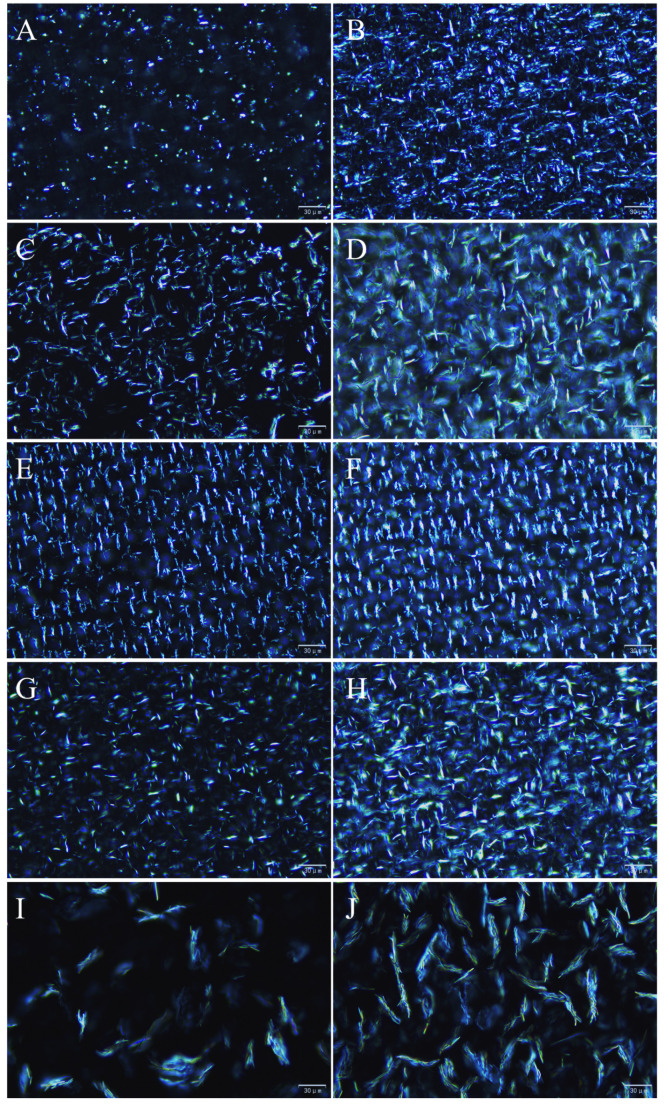
Polarized light microscopy images of CLW oleogels at critical (**A**) and 8 wt.% concentrations (**B**). Polarized light microscopy images of RBW oleogels at critical (**C**) and 8 wt.% concentrations (**D**). Polarized light microscopy images of CRW oleogels at critical (**E**) and 8 wt.% concentrations (**F**). Polarized light microscopy images of BW oleogels at critical (**G**) and 8 wt.% concentrations (**H**). Polarized light microscopy images of GMS oleogels at critical (**I**) and 8 wt.% concentrations (**J**). All images were taken at 40× magnification at 25 °C. Scale bar = 30 μm.

Figure 2 shows the polarized light micrographs of the oleogels formed with different gelling agents at critical and 8 wt.% concentrations. In the polarized light field, crystals are birefringent, whereas the liquid oil phase is optically isotropic and therefore black. As shown in Figure 2, the microstructures of the oleogels formed by different gelling agents vary considerably. The crystals in the CLW oleogel at the critical concentration were short rod-like structures of approximately 4 μm in length, which were uniformly scattered in the SO. With an increase in the CLW concentration, longer crystals (20–30 μm) in the shape of slender needles were observed. These long needle-like structures can form a good crystal matrix and interlock well at the intercrystalline interface to form organogels. The oleogel with 5 wt.% RBW contained crescent-shaped crystalline units, which were relatively sparsely distributed, and a tight gel network structure was not formed. When the amount of added RBW was 8 wt.%, flocculent crystallization occurred and the crystal distribution density increased. Bundled crystals with lengths of approximately 20 μm and a regular distribution were observed in the CRW oleogel. However, the crystals tended to be arranged in the same direction, which may be unfavorable for the formation of a three-dimensional network structure. A small number of fine needle-like crystals were observed at a BW concentration of 3 wt.%. In contrast, when the concentration of BW was 8 wt.%, the fine needle-like crystals formed clusters, and an interwoven three-dimensional network structure could be clearly observed, suggesting that this specific microstructure could provide control over the diffusion of volatile flavor substances. Additionally, at 8 wt.% gelator, the hazy appearance of RBW, CRW, and BW may be due to the formation of spherulitic-type structures made of assemblies of needles or platelets which are out of the focal plane [29,30]. The crystal structure of the GMS oleogel was feather-like, and the crystal length tended to increase with an increase in the amount of gelling agent. Compared with wax-based oleogels, the distribution of crystals in the GMS oleogels was sparser, resulting in numerous blank areas and the formation of a less dense network structure, which is consistent with the texture analysis results. It indicates that GMS oleogels were weaker than the plant wax-based systems at equivalent concentrations. The different microstructures of the various oleogels may have different effects on controlling the diffusion of volatile compounds.

### 3.3. Texture Analysis

**Figure 3:**
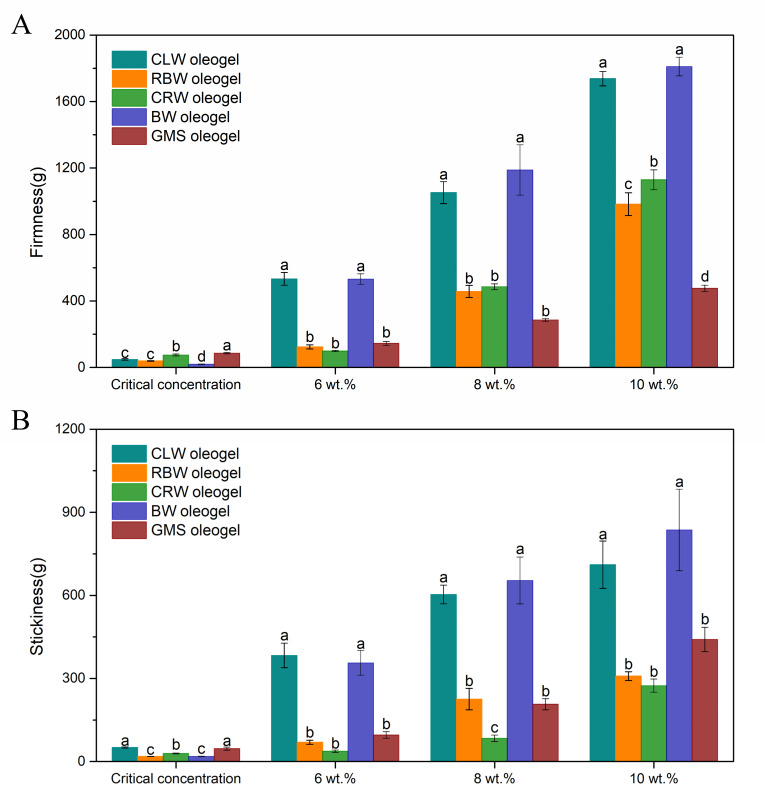
Firmness (**A**) and stickiness (**B**) of the oleogels at room temperature (~25 °C). Sample preparation and analyses were performed in triplicate, and the results were expressed as mean with error bar. Different letters represent statistical differences between values within each panel (*p* < 0.05).

In order to evaluate the feasibility of replacing solid fats with oleogels from various aspects, the texture parameters and thermodynamic properties of different oleogels were analyzed. The firmness depends on the density of a gel network structure, whereas the stickiness indicates its cohesiveness. A high density and cohesiveness provide a gel with structural integrity and resistance to external forces. As shown in Figure 3, the firmness of each wax-based oleogel sample was close to zero at the critical concentration, although there was no gravitational flow when the samples were inverted. At higher concentrations, the firmness and stickiness of each oleogel increased because the total crystalline solids increased, and the gel network structure became tighter (Figure 2). At a gelling agent concentration of 8 wt.%, the firmness of CLW and BW were 1051.71 and 1188.53 g, respectively, whereas those of RBW, CRW, and GMS were 457.39, 485.28, and 285.68 g, respectively. CLW and BW showed significantly higher firmness than the other waxes or GMS, which may be due to their lower critical concentrations and the formation of more crystalline solids at the same concentration. The formation of closed spaces within the oleogel is the basis for flavor retention. The harder the oleogel is, the stronger the oil binding property is. Therefore, the oil mobility within the crystalline network is limited, which may have a greater effect on the migration of flavor molecules.

### 3.4. Thermal Properties

**Figure 4:**
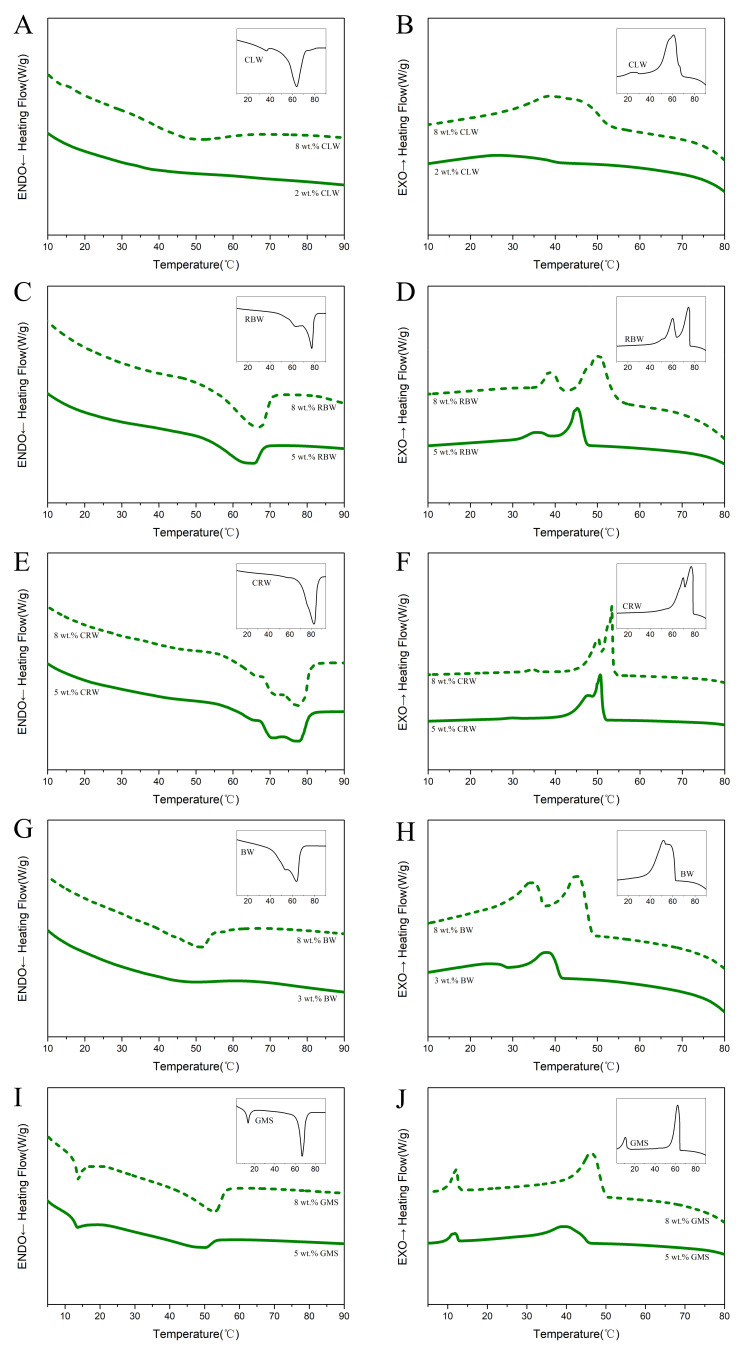
DSC thermograms during heating (**A**) and cooling (**B**) of CLW oleogels. DSC thermograms during heating (**C**) and cooling (**D**) of RBW oleogels. DSC thermograms during heating (**E**) and cooling (**F**) of CRW oleogels. DSC thermograms during heating (**G**) and cooling (**H**) of BW oleogels. DSC thermograms during heating (**I**) and cooling (**J**) of GMS oleogels.

Figure 4 shows the melting and crystallization behaviors of the oleogels formed with different gelling agents at critical and 8 wt.% concentrations as well as the thermal behavior of each gelling agent alone for comparison. Waxes are complex mixtures of multiple compounds with both major and minor components. The oleogels were obtained by diluting the gelling agents in SO, which caused the exothermic and endothermic peaks of the oleogel to be broadened and shifted to lower temperatures as compared to those of the gelling agent alone. In addition, the melting and crystallization behavior of the oleogel was determined by the main components of the gelling agent because of the dilution effect. As shown in Figure 4A,B, the CLW oleogel exhibited no significant exothermic or endothermic peaks at the critical concentration of 2 wt.%, which may be due to the low gelling agent concentration, less crystallization in the oleogel, or a nucleation rate too slow to be detected. Broad endothermic and exothermic peaks were observed at a CLW concentration of 8 wt.%, which are mainly related to the *n*-alkanes in CLW.

As shown in Figure 4D, two exothermic peaks were observed in the crystallization curve of the RBW oleogel, as in the case of RBW alone. For the 5 and 8 wt.% RBW oleogels, the first exothermic peak was located at 44.51 ± 0.56 °C and 49.38 ± 1.00 °C, respectively, whereas the second exothermic peak was located at 34.54 ± 0.19 °C and 38.28 ± 0.75 °C, respectively. The crystallization peaks gradually shifted toward higher temperatures as more RBW was added. In contrast, only one endothermic peak was observed during the melting of the RBW oleogel (Figure 4C), which may be because the constituents of RBW have similar melting temperatures. As shown in Figure 4E,F, the CRW oleogels exhibited complex melting and crystallization behaviors with multiple endothermic and exothermic peaks, indicating the presence of a series of different solids in CRW. In addition, the melting peaks of the CRW oleogels varied between 69.87 ± 0.35 °C and 77.72 ± 0.38 °C, which indicates that CRW has a higher melting temperature. Figure 4G,H show that the melting and crystallization behaviors of the BW oleogels with different concentrations of the gelling agent are similar. Two major broad exothermic peaks were observed, which are related to the major components (plant waxes) and minor components (hydrocarbons) in BW [31]. The onset gelation temperature (T_g_), peak temperature of crystallization (T_c_), and enthalpy change of crystallization (ΔH_c_) of the oleogel during the cooling phase increased with increasing BW content. As shown in Figure 4I,J, two endothermic and exothermic peaks were observed for the GMS oleogels, which are related to monopalmitin and monostearin in GMS. The peak temperatures (T_c_, T_m_) in the low-temperature region did not increase significantly when the gelling agent content was increased. In contrast, similar to the trend observed for the melting behavior, the endothermic peak in the high-temperature region shifted toward a higher temperature as the concentration of the gelling agent increased.

The enthalpy change reflects that the free energy is required to cause the state change of the material, with a larger enthalpy change indicating more spontaneous oleogel formation. As shown in Table 1, the type and amount of gelling agent influence the enthalpy change of crystallization (ΔH_c_) and the enthalpy change of melting (ΔH_m_) of the oleogels. In general, the ΔH_c_ and ΔH_m_ values tend to increase with an increase in the gelling agent concentration, which indicates that the arrangement of gelant molecules in the system is more organized and the spontaneity of oleogel formation increases. Moreover, for each oleogel, the ΔH_c_ value was higher than the ΔH_m_ value, and this hysteresis can be explained by the heat of dissolution of the gelling agent during heating. As reported by Abdallah et al. [32], the increase in temperature leads to network fracture, and the dissolution of solid materials as exothermic materials may inhibit the visualization of heat-absorbing melting events. The results of DSC showed that the melting points of CLW, BW and GMS oleogels were relatively close to butter (32–35 °C), which proved the feasibility of partial substitution of butter for preparing low-trans and low-saturated fatty acid foods. In contrast, the melting temperatures of RBW and CRW oleogels were too high, which might cause undesirable sensory experiences during consumption.

### 3.5. XRD Analysis

**Figure 5:**
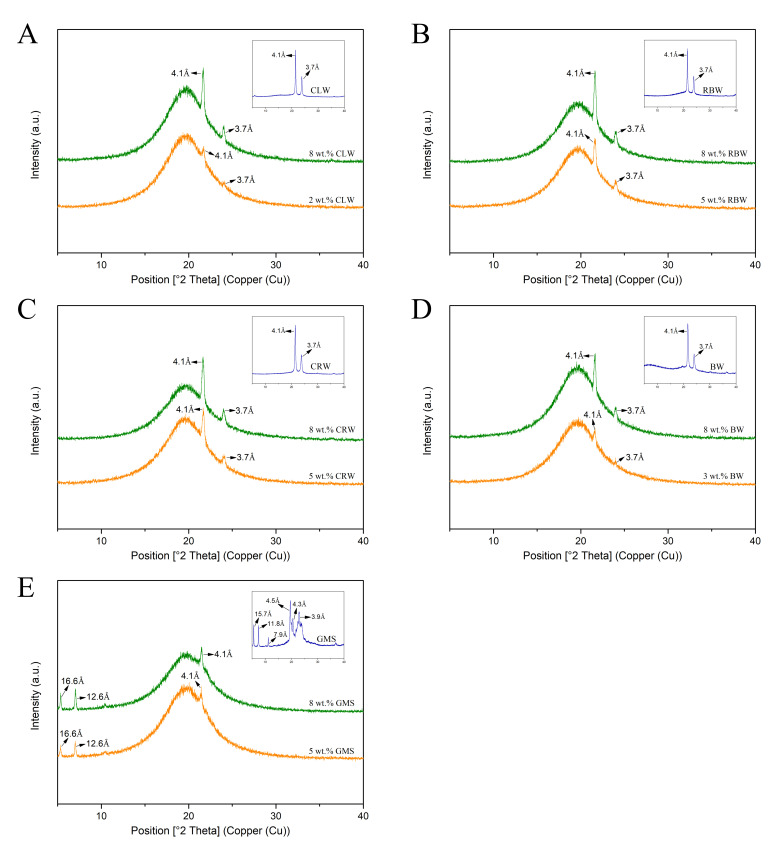
The X-ray diffractograms of oleogels formed by different gel factors at critical and 8 wt.% concentrations, (**A**) CLW oleogels; (**B**) RBW oleogels; (**C**) CRW oleogels; (**D**) BW oleogels; (**E**) GMS oleogels.

Polymorphism refers to the phenomenon in which a molecule (or atom) forms two or more different packing modes and crystal structures under different conditions. Polymorphism mainly depends on the arrangement of the molecules in the crystal lattice. Changes in the hydrocarbon chain stacking pattern and tilt angles in lipids can cause differences in crystal forms, each of which has a different short spacing (the distance between parallel acyl groups on triglycerides), which can be analyzed using XRD [33]. Figure 5 shows the XRD patterns of the oleogels formed with different gelling agents at critical and 8 wt.% concentrations, as well as those of the gelling agents alone for comparison. All the wax-based oleogels showed XRD patterns similar to that of the corresponding waxes, with two strong wide-angle diffraction peaks appearing at 4.1 and 3.7 Å, which indicates the orthorhombic perpendicular (O_⊥_) subcell packing (β’ morphology) [34]. Among the three different polymorphs of triglycerides (α, β, and β’), the β’ crystal form has the strongest plasticity, showing better spreadability and mouthfeel. The main diffraction peaks of GMS appeared at d = 15.7, 11.8, 7.9, 4.5, 4.3, and 3.9 Å, whereas those of the GMS oleogel were observed at d = 16.6, 12.6, and 4.1 Å, which correspond to the characteristic diffraction peaks of the α morphology with hexagonal symmetry. The peaks (T_c_, T_m_) observed in the low-temperature region during crystallization and cooling of the GMS oleogels (Figure 4I,J) are consistent with the results of Doan et al. [35]. The orientational order of the ‘zig-zag’ planes disappears, and the molecules can rotate around their long axis to form a hexagonally symmetric structure when the orthorhombic gelling agent undergoes a phase transition at a temperature close to its melting point. In addition, for different gelling agents, the intensity of the short-spacing peaks increased with an increase in the gelling agent concentration, indicating that the stacking pattern and polycrystalline structure of the oleogel molecules depend on the gelling agent concentration.

### 3.6. Release Kinetics of Flavor Compounds

The contents of 28 of the main flavor substances in SO were analyzed before and after storage (Table 2). Based on the retention effects in oleogels, these compounds can be divided into two categories. The first category includes 18 flavor substances with high saturated vapor pressures and low hydrophobic constants, which are extremely volatile in SO. After 60 d of open storage, 2-pentanone, 2-methylbutanal, pyrazine, 2-methylthiazole, 4-methylthiazole, and (5-methyl-2-furyl)methanol were depleted in SO, and the content of the other flavor substances also decreased significantly, resulting in a decline in SO quality and flavor characteristics. This behavior is consistent with the results of Chen et al. [36], who found that for an active ingredient with high vapor pressure, the diffusion rate often depends on the volatility of the substance, which is usually high. However, the addition of gelling agents slowed the release of these compounds in SO to varying degrees. This effect is due to the rigid crystal network, which traps the volatile substances within the internal phase and acts as a physical barrier. The CLW and BW oleogels were more effective in retaining such substances, which may be related to their higher textural parameters. The firmness of the CLW and BW oleogels was significantly higher than that of the oleogels with other waxes and monoglycerides (Figure 3A). These oleogels formed interwoven three-dimensional network structures (Figure 2B,H) inside, which can impede the mass transfer of flavor substances across the oil to the headspace, thereby inhibiting the loss of flavor substances. Yin et al. [37] showed that the sensory attributes “sweet”, “toasted”, “nutty”, “persistent”, “high-intense flavor” and “cooked sesame seed flavor” were drivers of liking. Pyrazines contributed to improve roasted flavors and increase consumer acceptance of sesame oil. Therefore, the retention of such compounds is of relevance.

The second category mainly includes compounds with low saturated vapor pressures or hydrophobic compounds. The changes in the contents of these compounds before and after storage were relatively small, and the retention effect in SO was better. Unlike the first category of compounds, the addition of a gelling agent promoted the release of compounds of the second category to a certain extent. This behavior may be due to the self-assembly of the gelling agent in SO to form hydrophilic structural domains [38], which can reduce the hydrophobicity of SO and increase the release of lipophilic volatiles.

To further investigate the effect of gelation on the controlled release behavior of the flavor substances in SO, the cumulative release rates of the flavor substances in different oleogels and SO were fitted using the zero-order release, first-order release, Higuchi model, Kormeyer–Peppas model, and Weibull equations (see Supplementary Materials Table S2). The model with the best fitting effect (highest correlation coefficient, R^2^) was the Weibull equation (R^2^ > 0.97), and the flavor release kinetics were analyzed using this model. During the open storage process, the flavor substances in the oil phase were released owing to the effect of internal and external concentration differences. Because the concentrations of the flavor substances in the samples were higher during the initial stage, the release rate was faster. At longer storage times, the concentrations of the flavor substances in the samples decreased, which decreased the driving force for release and consequently the release rate. Therefore, the dynamic law obtained from the Weibull equation is as follows: Q = 1 − exp[−(kt)^n^] (Table S2).

**Figure 6:**
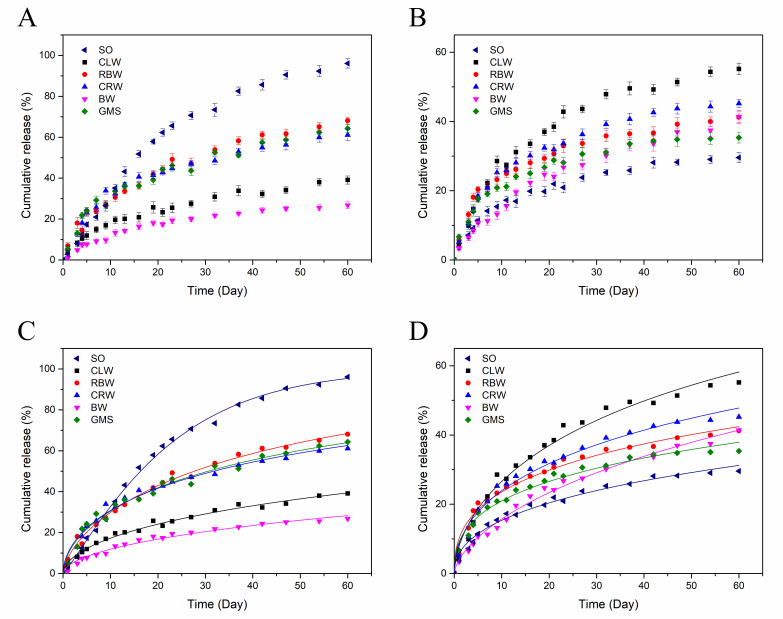
Dynamic release curves of 2-methylpyrazine (**A**) and nonanal (**B**). Weibull model fitting release curves of 2-methylpyrazine (**C**) and nonanal (**D**).

For each category of compounds with different retention effects in the oleogel, a representative example was selected to study the change in the cumulative release rate during storage for 60 d. The results showed that the release of the flavor compounds in the open environment followed the kinetic law of the Weibull equation and a high degree of fit was obtained. The first category of compounds was exemplified by 2-methylpyrazine, which is highly volatile in SO because of its high saturated vapor pressure. As shown in Figure 6A the cumulative release rate of 2-methylpyrazine reached 96.08% after 60 d of storage, implying that it is quickly depleted from SO. The addition of the gelling agent significantly (*p* < 0.05) reduced the release rate and quantity of 2-methylpyrazine. The CLW and BW oleogels had the best retention effects on 2-methylpyrazine, with cumulative release rates of 39.09% and 26.76%, respectively, at the end of the storage. The RBW, CRW, and GMS oleogels exhibited smaller retention effects, with cumulative release rates of 68.12%, 61.13%, and 64.33%, respectively. The release rate constant, k, indicates the rapidity of release, with a larger the k value corresponding to faster release. As shown in Table 3, the largest k value for 2-methylpyrazine was observed in SO (0.0445). In contrast, the k values for the RBW, CRW, and GMS oleogels were 0.0207, 0.0162, and 0.0173, respectively. The k values for the CLW and BW oleogels were 0.0047 and 0.0022, respectively, indicating a significant decrease in the release rate, which is consistent with the cumulative release rates of 2-methylpyrazine after storage for 60 d. The n value in the Weibull equation corresponds to the release mechanism. The n values for 2-methylpyrazine in the oleogels ranged from 0.5402 to 0.6572, indicating a release mechanism between restricted diffusion and a first-order reaction. However, the n value for 2-methylpyrazine in SO was 1.1422, indicating controlled release with an initial induction time.

The second category of compounds was exemplified by nonanal. As shown in Figure 6B, after 60 d of open storage, nonanal had the best retention effect in SO, with a cumulative release rate of only 29.58% and a k value of 0.0019. For the GMS oleogel, the cumulative release rate was 35.37% with a k value of 0.0025. When the release process was finished, there was no significant difference in the nonanal content in the RBW, CRW, and BW oleogels, and the cumulative release rate was 41.23–45.22%. Unlike the retention effect observed for the first type of compound, the addition of CLW promoted the release of nonanal. It is speculated that the main *n*-alkane components of CLW may compete with the binding sites for the hydrophobic flavor substances, thus decreasing the stability. In addition, the n values for nonanal in SO and the oleogels were 0.3943–0.6352, which corresponds to the release mechanism between restricted diffusion and a first-order reaction. In general, the addition of gelling agents promoted the release of the second type of compounds to varying degrees, but due to their low saturated vapor pressures, losses after storage is relatively low and has little effect on the overall flavor. Overall, the BW oleogel exhibited a good retention effect on flavor molecules with different physicochemical properties. Thus, this gelling agent can be used to establish a controllable flavor delivery system and realize SO flavor stabilization and retention technologies.

## 4. Conclusions

In this study, sesame-oil-based oleogels were prepared with biological waxes and monoglycerides as gelling agents to form controllable flavor delivery systems. The obtained results showed clearly that sesame oil gelation enables controlled release of flavor substances, and that this retention is more significant for volatiles with high saturated vapor pressures and low hydrophobic constants. This is due to the rigid crystal network formed by gelation, which traps the volatiles within the internal phase and acts as a physical barrier. The release of flavor substances in SO during storage can be described by the Weibull equation kinetic model. BW oleogel is the most effective gel system for the controlled release of flavor substances, with good retention of flavor substances with different physicochemical properties. This is related to the high firmness of the BW oleogel and the interwoven three-dimensional network structure inside. DSC and XRD results showed that the wax-based oleogels had good plasticity and suitable melting temperatures, which confirmed the feasibility of these systems for replacing solid fats. We hope that these findings will assist in the design and construction of gel systems for vegetable oils with different flavor profiles to achieve the controlled release of specific flavors.

## Figures and Tables

**Table 1:** Thermodynamic parameters of oleogels formed by different types of gelling agent.

Sample	Crystallization Process				Melting Process			
	T_g_ (°C)	T_c1_ (°C)	T_c2_ (°C)	ΔH_c_ (J/g)	T_e_ (°C)	T_m1_ (°C)	T_m2_ (°C)	ΔH_m_ (J/g)
2 wt.% CLW	ND ^i^	ND ^h^	ND ^g^	ND ^i^	ND ^i^	ND ^i^	ND ^d^	ND ^i^
8 wt.% CLW	52.73 ± 0.09 ^b^	41.34 ± 0.10 ^f^	ND ^g^	7.68 ± 0.04 ^e^	58.69 ± 0.09 ^e^	46.89 ± 0.18 ^f^	ND ^d^	7.23 ± 0.12 ^d^
5 wt.% RBW	47.54 ± 0.25 ^f^	44.51 ± 0.56 ^e^	34.54 ± 0.19 ^d^	8.35 ± 0.18 ^d^	70.04 ± 0.72 ^d^	63.28 ± 0.38 ^d^	ND ^d^	7.90 ± 0.11 ^c^
8 wt.% RBW	53.52 ± 0.31 ^a^	49.38 ± 1.00 ^c^	38.28 ± 0.75 ^c^	12.34 ± 0.02 ^a^	71.26 ± 0.06 ^c^	65.95 ± 0.10 ^c^	ND ^d^	12.11 ± 0.12 ^a^
5 wt.% CRW	51.60 ± 0.23 ^c^	50.95 ± 0.27 ^b^	47.94 ± 0.09 ^b^	7.02 ± 0.03 ^f^	79.97 ± 0.14 ^b^	69.87 ± 0.35 ^b^	76.80 ± 0.08 ^a^	3.90 ± 0.05 ^g^
8 wt.% CRW	53.71 ± 0.02 ^a^	53.30 ± 0.04 ^a^	49.81 ± 0.17 ^a^	11.41 ± 0.03 ^b^	81.26 ± 0.55 ^a^	71.21 ± 0.09 ^a^	77.72 ± 0.38 ^a^	6.12 ± 0.11 ^e^
3 wt.% BW	41.31 ± 0.03 ^h^	38.66 ± 0.07 ^g^	23.28 ± 0.02 ^e^	1.94 ± 0.04 ^h^	53.00 ± 0.14 ^h^	45.01 ± 0.01 ^g^	ND ^d^	1.76 ± 0.02 ^h^
8 wt.% BW	48.45 ± 0.01 ^e^	45.59 ± 0.19 ^de^	34.13 ± 0.11 ^d^	7.02 ± 0.01 ^f^	54.68 ± 0.12 ^g^	49.76 ± 0.42 ^e^	ND ^d^	4.98 ± 0.04 ^f^
5 wt.% GMS	44.58 ± 0.03 ^g^	40.20 ± 0.02 ^f^	11.34 ± 0.08 ^f^	4.33 ± 0.03 ^g^	52.95 ± 0.05 ^h^	12.84 ± 0.06^h^	47.18 ± 0.11 ^c^	4.11 ± 0.04 ^g^
8 wt.% GMS	49.52 ± 0.04 ^d^	46.67 ± 0.06 ^d^	11.92 ± 0.10 ^f^	9.86 ± 0.07 ^c^	56.59 ± 0.10 ^f^	13.03 ± 0.08 ^h^	51.58 ± 0.25 ^b^	9.74 ± 0.05 ^b^

Each sample was analyzed in triplicate, and the results were expressed as mean ± standard deviation (x ± SD). Values in the same column with different superscript letters are significantly different at *p* < 0.05. T_g_, onset gelation temperature; T_c1_, first peak temperature of crystallization; T_c2_, second peak temperature of crystallization; ΔH_c_, enthalpy change of crystallization; T_e_, melting complete temperature; T_m1_, first peak temperature in the heating phase; T_m2_, second peak temperature in the heating phase; ΔH_m_, enthalpy change of melting. ND = not detectable.

**Table 2:** Changes in the content of the main volatile compounds in samples before and after storage (μg/kg, mean ± SD).

Volatiles	Aroma Description	Log *p*	Vapor Pressure (mmHg at 25 °C)	Initial Concentration (μg/kg)	Post-Storage Concentrations (μg/kg)					
					CLW	RBW	CRW	BW	GMS	SO
**Significant retention effect**										
2-Pentanon	ethereal, fruity	0.9	38.6 ± 0.2	1920 ± 114	1985 ± 46 ^a^	419 ± 46 ^c^	345 ± 19 ^c^	2013 ± 76 ^a^	606 ± 37 ^b^	Nd ^d^
2-Methylbutanal	flowers, grass	1.25	49.3 ± 0.2	2151 ± 136	1907 ± 54 ^b^	662 ± 8 ^c^	379 ± 40 ^d^	2142 ± 58 ^a^	427 ± 79 ^d^	Nd ^e^
Pyrazine	pungent, sweet, corn, nutty	−0.28	19.7 ± 0.2	4891 ± 367	2787 ± 140 ^b^	1201 ± 56 ^d^	1583 ± 86 ^c^	3291 ± 180 ^a^	1376 ± 85 ^cd^	Nd ^e^
Hexanal	fatty-green, grassy	1.97	10.9 ± 0.2	2171 ± 126	2144 ± 59 ^a^	1703 ± 15 ^b^	1779 ± 39 ^b^	2184 ± 36 ^a^	1702 ± 143 ^b^	1127 ± 68 ^c^
2-Methylthiazole	nutty, green	1.10	12.9 ± 0.2	572 ± 19	451 ± 29 ^b^	Nd ^d^	253 ± 20 ^c^	569 ± 23 ^a^	289 ± 19 ^c^	Nd ^d^
4-Methylthiazole	nutty, green	0.90	10.0 ± 0.2	3242 ± 241	2442 ± 137 ^a^	680 ± 89 ^b^	883 ± 40 ^b^	2450 ± 22 ^a^	930 ± 162 ^b^	Nd ^c^
2-Methylpyrazine	nutty, cocoa-like	0.18	9.7 ± 0.2	43,839 ± 1291	26,702 ± 532 ^b^	13,976 ± 243 ^d^	17,040 ± 445 ^c^	32,108 ± 611 ^a^	15,637 ± 376 ^c^	1718 ± 78 ^e^
Furfural	almond-like	0.73	2.2 ± 0.3	13,949 ± 67	7431 ± 25 ^b^	2148 ± 219 ^d^	1722 ± 28 ^de^	9332 ± 214 ^a^	4348 ± 240 ^c^	1356 ± 69 ^e^
2-Furanmethanol	faint, burning odor	0.20	1.0 ± 0.3	15,654 ± 650	6936 ± 316 ^b^	3718 ± 81 ^c^	2583 ± 252 ^d^	9267 ± 282 ^a^	3957 ± 92 ^c^	1012 ± 47 ^e^
3-Methylpyridine	green	1.19	6.7 ± 0.3	3109 ± 352	1616 ± 43 ^cd^	2107 ± 66 ^ab^	1839 ± 55 ^bc^	2322 ± 115 ^a^	1329 ± 130 ^d^	1675 ± 65 ^c^
2,4-Dimethylthiazole	meat, cocoa-like	1.56	4.3 ± 0.3	1489 ± 47	947 ± 34 ^b^	628 ± 8 ^d^	721 ± 18 ^cd^	1354 ± 70 ^a^	790 ± 12 ^c^	275 ± 9 ^e^
1-(2-Furyl)ethanone	coffee-like	0.52	0.8 ± 0.3	2131 ± 207	1279 ± 22 ^b^	824 ± 80 ^de^	1035 ± 32 ^cd^	1746 ± 77 ^a^	1187 ± 112 ^bc^	737 ± 15 ^e^
2,5-Dimethylpyrazine	nutty, coffee-like	0.64	4.0 ± 0.2	37,933 ± 2490	24772 ± 243 ^b^	16,760 ± 434 ^d^	21,229 ± 1096 ^c^	33103 ± 797 ^a^	21,964 ± 1712 ^bc^	12,523 ± 541 ^e^
2-Ethylpyrazine	nutty, peanut butter	0.71	4.0 ± 0.3	6609 ± 474	3791 ± 163 ^b^	2141 ± 33 ^d^	2762 ± 58 ^c^	4650 ± 98 ^a^	2898 ± 350 ^c^	1566 ± 85 ^e^
2,3-Dimethylpyrazine	nutty, cocoa-like	0.64	3.4 ± 0.3	9595 ± 544	5026 ± 123 ^b^	3528 ± 192 ^de^	4205 ± 49 ^cd^	6302 ± 51 ^a^	4656 ± 394 ^bc^	3207 ± 229 ^e^
2-Vinylpyrazine	coffee-like	0.58	3.3 ± 0.3	2822 ± 178	1383 ± 53 ^b^	960 ± 14 ^cd^	1118 ± 88 ^c^	1902 ± 37 ^a^	1332 ± 32 ^b^	914 ± 22 ^d^
(5-Methyl-2-furyl)methanol	sweet caramel-like	0.66	0.6 ± 0.4	3488 ± 129	564 ± 33 ^b^	Nd ^c^	Nd ^c^	912 ± 68 ^a^	Nd ^c^	Nd ^c^
5-Methyl-2-furaldehyde	spicy-sweet, slightly caramellic	1.19	0.6 ± 0.3	19,949 ± 1301	9361 ± 482 ^d^	13,288 ± 311 ^c^	15,386 ± 224 ^b^	17,974 ± 593 ^a^	17,995 ± 264 ^a^	14383 ± 434 ^bc^
**No significant retention effect**										
2-Ethyl-6-methylpyrazine	roasted baked potato	1.17	2.0 ± 0.3	11,203 ± 492	5869 ± 71 ^bc^	5209 ± 148 ^c^	6547 ± 162 ^b^	8711 ± 161 ^a^	8034 ± 253 ^a^	8424 ± 334 ^a^
2,3,5-Trimethylpyrazine	roasted nut, baked potato	1.10	1.7 ± 0.3	26,542 ± 1612	15,006 ± 169 ^c^	12,903 ± 275 ^d^	17,151 ± 109 ^b^	21,770 ± 555 ^a^	20,495 ± 870 ^a^	21,616 ± 754 ^a^
1H-Pyrrole-2-carbaldehyde	grassy	0.64	0.1 ± 0.4	18,461 ± 877	9879 ± 34 ^d^	10,000 ± 567 ^d^	12,381 ± 459 ^c^	14,916 ± 454 ^b^	14,085 ± 219 ^b^	17815 ± 667 ^a^
2-Acetylpyrazine	nutty, popcorn, breadcrust	0.16	0.2 ± 0.4	16,477 ± 398	8040 ± 116 ^d^	7261 ± 240 ^d^	9427 ± 119 ^c^	11,794 ± 405 ^b^	12,424 ± 231 ^b^	13,373 ± 287 ^a^
1-(2-Pyridinyl)ethanone	tobacco, heavy-oily-fatty	0.87	0.5 ± 0.4	11,234 ± 701	5926 ± 55 ^d^	5122 ± 152 ^e^	8629 ± 209 ^c^	9433 ± 337 ^b^	9076 ± 188 ^bc^	11039 ± 148 ^a^
3-Ethyl-2,5-dimethylpyrazine	nutty, coffee-like	1.63	1.2 ± 0.3	23,354 ± 1357	10,051 ± 435 ^d^	9395 ± 83 ^d^	12,325 ± 234 ^c^	14,329 ± 478 ^b^	15,420 ± 349 ^b^	18,050 ± 236 ^a^
2-Methoxyphenol	smoke, somewhat medicinal	1.19	0.2 ± 0.4	38,612 ± 1786	21,634 ± 691 ^d^	22,497 ± 142 ^d^	27,067 ± 697 ^c^	31,870 ± 1387 ^b^	34,329 ± 251 ^b^	37,909 ± 605 ^a^
Nonanal	fruity	3.56	0.5 ± 0.4	11,488 ± 504	5147 ± 186 ^d^	6751 ± 169 ^c^	6293 ± 157 ^c^	6731 ± 212 ^c^	7425 ± 140 ^b^	8090 ± 167 ^a^
2-Acetyl-6-methylpyrazine	nutty	0.62	0.1 ± 0.5	26,345 ± 1182	10,545 ± 72 ^d^	13,628 ± 108 ^c^	13,967 ± 173 ^c^	15,098 ± 207 ^b^	15,554 ± 571 ^b^	18,861 ± 510 ^a^
2-Methoxy-4-vinylphenol	spicy, roasted peanut	1.93	0.0 ± 0.5	43,737 ± 1419	16,196 ± 162 ^d^	20,740 ± 518 ^bc^	23,432 ± 393 ^b^	20,378 ± 284 ^c^	21,890 ± 799 ^bc^	27,162 ± 1896 ^a^

Values in the same row with different superscript letters are significantly different at *p* < 0.05. Nd = not detectable.

**Table 3:** Fitting results of Weibull equations of 2-methylpyrazine and nonanal in different samples.

Sample	2-Methylpyrazine			Nonanal		
	k	n	R^2^	k	n	R^2^
SO	0.0445	1.1422	0.9966	0.0019	0.4518	0.9799
CLW oleogel	0.0047	0.5424	0.9877	0.0132	0.5791	0.9804
RBW oleogel	0.0207	0.6572	0.9898	0.0038	0.4019	0.9770
CRW oleogel	0.0162	0.5483	0.9820	0.0069	0.4840	0.9799
BW oleogel	0.0022	0.5402	0.9763	0.0062	0.6352	0.9897
GMS oleogel	0.0173	0.5759	0.9795	0.0025	0.3943	0.9750

k, the release rate constant; n, the release mechanism constant; R^2^, coefficient of determination

## Data Availability

All data included in this study are available upon request by contact with the corresponding author.

## Supplementary Materials

The following are available online at https://www.mdpi.com/article/10.3390/foods10081828/s1, Table S1: Minimum quantity (wt.%) required for SO gelation and composition of gelling agents, Table S2: Dynamics equation.

## References

[1] Wan Y., Li H., Fu G., Chen X., Chen F., Xie M. (2015). The relationship of antioxidant components and antioxidant activity of sesame seed oil. J. Sci. Food Agric..

[2] Konsoula Z., Liakopoulou-Kyriakides M. (2010). Effect of endogenous antioxidants of sesame seeds and sesame oil to the thermal stability of edible vegetable oils. LWT Food Sci. Technol..

[3] Kumar C.M., Singh S.A. (2015). Bioactive lignans from sesame (*Sesamum indicum* L.): Evaluation of their antioxidant and antibacterial effects for food applications. J. Food Sci. Technol..

[4] Varelas C.G., Dixon D.G., Steiner C.A. (1995). Zero-order release from biphasic polymer hydrogels. J. Control. Release.

[5] Gibaldi M., Feldman S. (1967). Establishment of sink conditions in dissolution rate determinations. Theoretical considerations and application to nondisintegrating dosage forms. J. Pharm. Sci..

[6] Higuchi T. (1961). Rate of Release of Medicaments from Ointment Bases Containing Drugs in Suspension. J. Pharm. Sci..

[7] Neoh T.-L., Yoshii H., Furuta T. (2006). Encapsulation and Release Characteristics of Carbon Dioxide in α-Cyclodextrin. J. Incl. Phenom. Macrocycl. Chem..

[8] Pușcaș A., Mureșan V., Socaciu C., Muste S. (2020). Oleogels in Food: A Review of Current and Potential Applications. Foods.

[9] Patel A.R. (2017). A colloidal gel perspective for understanding oleogelation. Curr. Opin. Food Sci..

[10] Meng Z., Qi K., Guo Y., Wang Y., Liu Y. (2018). Macro-micro structure characterization and molecular properties of emulsion-templated polysaccharide oleogels. Food Hydrocoll..

[11] Chopin-Doroteo M., Morales-Rueda J.A., Dibildox-Alvarado E., Charó-Alonso M.A., de la Peña-Gil A., Toro-Vazquez J.F. (2011). The Effect of Shearing in the Thermo-mechanical Properties of Candelilla Wax and Candelilla Wax–Tripalmitin Organogels. Food Biophys..

[12] Troya F., Lerma-García M.J., Herrero-Martínez J.M., Simó-Alfonso E.F. (2015). Classification of vegetable oils according to their botanical origin using n-alkane profiles established by GC–MS. Food Chem..

[13] McGill A.S., Moffat C.F., Mackie P.R., Cruickshank P. (1993). The composition and concentration of n-alkanes in retail samples of edible oils. J. Sci. Food Agric..

[14] Moreda W., Pérez-Camino M.C., Cert A. (2001). Gas and liquid chromatography of hydrocarbons in edible vegetable oils. J. Chromatogr. A.

[15] Giuffrè A.M. (2021). n-Alkanes and n-Alkenes in Virgin Olive Oil from Calabria (South Italy): The Effects of Cultivar and Harvest Date. Foods.

[16] Giuffrè A.M. (2021). The effect of cultivar and harvest season on the n-alkane and the n-alkene composition of virgin olive oil. Eur. Food Res. Technol..

[17] Goh S.H., Gee P.T. (1986). Noncarotenoid hydrocarbons in palm oil and palm fatty acid distillate. J. Am. Oil Chem. Soc..

[18] Herchi W., Saoussem H., Rochut S., Boukhchina S., Kallel H., Pepe C. (2009). Characterization and Quantification of the Aliphatic Hydrocarbon Fraction during Linseed Development (*Linum usitatissimum* L.). J. Agric. Food Chem..

[19] Chen C.H., Terentjev E.M. (2009). Aging and Metastability of Monoglycerides in Hydrophobic Solutions. Langmuir.

[20] Yılmaz E., Öğütcü M., Yüceer Y.K. (2015). Physical Properties, Volatiles Compositions and Sensory Descriptions of the Aromatized Hazelnut Oil-Wax Organogels. J. Food Sci..

[21] Öğütcü M., Yılmaz E., Güneşer O. (2015). Influence of Storage on Physicochemical and Volatile Features of Enriched and Aromatized Wax Organogels. J. Am. Oil Chem. Soc..

[22] Toro-Vazquez J.F., Morales-Rueda J.A., Dibildox-Alvarado E., Charó-Alonso M., Alonzo-Macias M., González-Chávez M.M. (2007). Thermal and Textural Properties of Organogels Developed by Candelilla Wax in Safflower Oil. J. Am. Oil Chem. Soc..

[23] Morales-Rueda J.A., Dibildox-Alvarado E., Charó-Alonso M.A., Weiss R.G., Toro-Vazquez J.F. (2009). Thermo-mechanical properties of candelilla wax and dotriacontane organogels in safflower oil. Eur. J. Lipid Sci. Technol..

[24] Hwang H.-S., Kim S., Singh M., Winkler-Moser J.K., Liu S.X. (2012). Organogel Formation of Soybean Oil with Waxes. J. Am. Oil Chem. Soc..

[25] Abdallah D.J., Weiss R.G. (2000). n-Alkanes Gel n-Alkanes (and Many Other Organic Liquids). Langmuir.

[26] Maia M., Nunes F.M. (2013). Authentication of beeswax (*Apis mellifera*) by high-temperature gas chromatography and chemometric analysis. Food Chem..

[27] Wijarnprecha K., Aryusuk K., Santiwattana P., Sonwai S., Rousseau D. (2018). Structure and rheology of oleogels made from rice bran wax and rice bran oil. Food Res. Int..

[28] Dassanayake L.S.K., Kodali D.R., Ueno S., Sato K. (2009). Physical Properties of Rice Bran Wax in Bulk and Organogels. J. Am. Oil Chem. Soc..

[29] Doan C.D., Tavernier I., Okuro P.K., Dewettinck K. (2018). Internal and external factors affecting the crystallization, gelation and applicability of wax-based oleogels in food industry. Innov. Food Sci. Emerg. Technol..

[30] Blake A.I., Marangoni A.G. (2015). Plant wax crystals display platelet-like morphology. Food Struct..

[31] Doan C.D., To C.M., De Vrieze M., Lynen F., Danthine S., Brown A., Dewettinck K., Patel A.R. (2017). Chemical profiling of the major components in natural waxes to elucidate their role in liquid oil structuring. Food Chem..

[32] Abdallah D.J., Lu L., Weiss R.G. (1999). Thermoreversible Organogels from Alkane Gelators with One Heteroatom. Chem. Mater..

[33] Clarkson C.E., Malkin T. (1934). 139. Alternation in long-chain compounds. Part II. An X-ray and thermal investigation of the triglycerides. J. Chem. Soc..

[34] Yılmaz E., Öǧütcü M., Arifoglu N. (2015). Assessment of Thermal and Textural Characteristics and Consumer Preferences of Lemon and Strawberry Flavored Fish Oil Organogels. J. Oleo Sci..

[35] Doan C.D., Tavernier I., Bin Sintang M.D., Danthine S., Van de Walle D., Rimaux T., Dewettinck K. (2017). Crystallization and Gelation Behavior of Low- and High Melting Waxes in Rice Bran Oil: A Case-Study on Berry Wax and Sunflower Wax. Food Biophys..

[36] Chen X.-W., Chen Y.-J., Wang J.-M., Guo J., Yin S.-W., Yang X.-Q. (2017). Tunable volatile release from organogel-emulsions based on the self-assembly of β-sitosterol and γ-oryzanol. Food Chem..

[37] Yin W., Washington M., Ma X., Yang X., Lu A., Shi R., Zhao R., Wang X. (2020). Consumer acceptability and sensory profiling of sesame oils obtained from different processes. Grain Oil Sci. Technol..

[38] Chen X.-W., Guo J., Wang J.-M., Yin S.-W., Yang X.-Q. (2016). Controlled volatile release of structured emulsions based on phytosterols crystallization. Food Hydrocoll..

