# Supplementary material for: Controlled Release of Flavor Substances from Sesame-Oil-Based Oleogels Prepared Using Biological Waxes or Monoglycerides
Source: Foods. 2021 Aug 7;10(8):1828. doi: 10.3390/foods10081828 (PMC8392103; doi:10.3390/foods10081828)
Supplement: Supplementary file 1 [file foods-10-01828-s001.zip › foods-1322305-supplementary.pdf]

Supplementary Materials

Controlled release of flavour substances from sesame-oil-based oleogels prepared using biological waxes or monoglycerides

Min Pang [1,2,*], Lulu Cao [1,2], Shengmei Kang [1,2], Shaotong Jiang [1,2], Lili Cao [1,2,*]

[1] School of Food Science and Bioengineering, Hefei University of Technology, Hefei, 230009, P. R. China

[2] Key Laboratory for Agricultural Products Processing of Anhui Province, Hefei, 230009, P. R. China

***Corresponding author:**

Min Pang, pangmin@hfut.edu.cn

Lili Cao, lilycao504@hfut.edu.cn

Table S1. Minimum quantity (wt.%) required for SO gelation and composition of gelling agents.

Gelling agent	critical concentrations (wt.%)	Composition
Candelilla wax	2	*n*-alkanes (49-50%, mainly C29-C33), esters of acids and alcohols with even-numbered carbon chains (20-29%, mainly C28–34), resins (12-14%, mainly triterpenoid esters), free acids (7-9%) [22-24]
Rice bran wax	5	Esters derived from long-chain saturated fatty acids (C24 and C22) and long-chain saturated fatty alcohols (C28–C34) [27]
Carnauba wax	5	Aliphatic and aromatic esters (84–85%, including 40% aliphatic esters, 13% ω-hydroxy esters, and 8% cinnamic aliphatic diesters), free fatty acids (3-3.5%), alcohols (2-3%), lactides (2-3%), hydrocarbons (1-3%), resins (4-6%), moisture and inorganic residues (0.5-1%) [28]
Beeswax	3	odd-numbered hydrocarbons (40.1%, mainly heptacosane), even-numbered hydrocarbons (4.0%), odd-numbered monounsaturated hydrocarbons (12.2%), fatty acid esters combined with long-chain alcohols (43.7%), and free wax acids [26]
Glycerol monostearate	5	Monostearin (84.5%), monopalmitin (12.2%)

Table S2. Dynamics equation.

Dynamic mode	Fitting equation
Zero-order equation	$Q = Q_0 + kt$
First-order equation	$Q = 1 - \exp(-kt)$
Higuchi equation	$Q = kt^{0.5}$
Korsmeryer-Peppas equation	$Q = kt^n$
Weibull equation	$Q = 1 - \exp[-(kt)^n]$

Q, the cumulative release rate of flavor components at time t; k, the release rate constant; t, the storage time; n, the release mechanism constant.
